# Implementing pelvic floor muscle training in women's childbearing years: A critical interpretive synthesis of individual, professional, and service issues

**DOI:** 10.1002/nau.24256

**Published:** 2019-12-17

**Authors:** Victoria E. Salmon, E. J. C Hay‐Smith, Rachel Jarvie, Sarah Dean, Rohini Terry, Helena Frawley, Eivor Oborn, Susan E. Bayliss, Debra Bick, Clare Davenport, Christine MacArthur, Mark Pearson

**Affiliations:** ^1^ College of Medicine and Health University of Exeter Exeter UK; ^2^ Department of Women's and Children's Health, Dunedin School of Medicine University of Otago Dunedin New Zealand; ^3^ Department of Physiotherapy, School of Primary and Allied Health Care Monash University Melbourne Australia; ^4^ Warwick Business School University of Warwick Coventry UK; ^5^ Institute of Applied Health Research University of Birmingham Birmingham UK; ^6^ Warwick Clinical Trials Unit University of Warwick Coventry UK; ^7^ Wolfson Palliative Care Research Centre, Institute for Clinical & Applied Health Research, Hull York Medical School University of Hull Heslington UK

**Keywords:** antenatal education, critical interpretive synthesis, implementation, maternity services, pelvic floor muscle exercise, pelvic floor muscle training, urinary incontinence

## Abstract

**Aims:**

Antenatal pelvic floor muscle training (PFMT) may be effective for the prevention and treatment of urinary and fecal incontinence both in pregnancy and postnatally, but it is not routinely implemented in practice despite guideline recommendations. This review synthesizes evidence that exposes challenges, opportunities, and concerns regarding the implementation of PFMT during the childbearing years, from the perspective of individuals, healthcare professionals (HCPs), and organizations.

**Methods:**

Critical interpretive synthesis of systematically identified primary quantitative or qualitative studies or research syntheses of women's and HCPs attitudes, beliefs, or experiences of implementing PFMT.

**Results:**

Fifty sources were included. These focused on experiences of postnatal urinary incontinence (UI) and perspectives of individual postnatal women, with limited evidence exploring the views of antenatal women and HCP or wider organizational and environmental issues. The concept of agency (people's ability to effect change through their interaction with other people, processes, and systems) provides an over‐arching explanation of how PFMT can be implemented during childbearing years. This requires both individual and collective action of women, HCPs, maternity services and organizations, funders and policymakers.

**Conclusion:**

Numerous factors constrain women's and HCPs capacity to implement PFMT. It is unrealistic to expect women and HCPs to implement PFMT without reforming policy and service delivery. The implementation of PFMT during pregnancy, as recommended by antenatal care and UI management guidelines, requires policymakers, organizations, HCPs, and women to value the prevention of incontinence throughout women's lives by using low‐risk, low‐cost, and proven strategies as part of women's reproductive health.

## 1 INTRODUCTION

Pregnancy and childbirth are important risk factors for urinary incontinence (UI).^1^ Prevalence rates of UI at 30 weeks gestation have been reported as 31% in nulliparous women and 42% in parous women.^2^ Postpartum prevalence rates range from 30% in the first 3 months to 47% in the first 12 months.^3^ Three quarters of women reporting UI at 3 months after giving birth may still experience symptoms at least 12 years later.^4^ Incontinence places a large burden on women's physical, mental and social quality of life,^5^ with associated pressure on healthcare resources and wider societal costs.^6^ In the UK, the importance of preventative strategies has been recently highlighted in relation to safety,^7^ the attainment of reproductive health,^8^ and as a key component of the maternity service model envisaged in *Better Births*.^9^

Pelvic floor muscle training (PFMT) is “exercise to improve pelvic floor muscle strength, endurance, power, relaxation or a combination of these”.^10^ Training involves teaching performance of a correct voluntary pelvic floor muscle contraction (PFMC), individualized prescription of sufficient exercise dose (frequency, intensity, duration) to achieve desired changes in muscle physiology (for example, hypertrophy) and support for adherence to the prescribed treatment.^11^ PFMT may be effective for the prevention and treatment of urinary and fecal incontinence in pregnant and postnatal women, with those randomized to PFMT with supervision having 62% lower risk of reporting UI in late pregnancy and 29% three to 6 months after delivery.^12^ National and international guidelines recommend a population‐based approach, offering all pregnant women PFMT, regardless of continence status, to prevent antenatal and postnatal UI.^13^, ^14^, ^15^ The UK National Institute of Health and Care Excellence (NICE) antenatal care guidelines recommend providing pregnant women with information about PFMT at their booking appointment, with an opportunity to discuss issues and ask questions.^15^ International guidance goes further to recommend a supervised and intensive strengthening PFMT program with regular healthcare professional (HCP) contact.^13^ However, PFMT instruction in pregnancy–with fidelity to the evidence that informed the recommendations–is not routinely implemented in practice.^16^, ^17^, ^18^ In this review, implementation refers to the individual, professional, interprofessional, and organizational processes of putting PFMT into clinical practice. At the level of the service user (woman), the implementation process is more akin to uptake (engagement and participation), however broadly speaking, “implementation” can be considered to embrace all levels.

This review addresses the gap in knowledge about the challenges and opportunities for population‐level implementation of PFMT in routine maternity services so that research and services can proactively address implementation issues.

## 2 METHODS

We used critical interpretive synthesis,^19^ an interpretive approach to systematic review,^20^ to synthesize evidence that exposes challenges, opportunities, and concerns regarding the implementation of PFMT during the childbearing years, from the perspective of individuals, HCPs, and organizations.

Sources for inclusion were identified through structured database searches (see Table 1) supplemented by purposive searches. Sources were included if they reported direct experiences, attitudes, beliefs or behaviors of women or HCPs regarding PFMT, and presented either a contemporary view of PFMT for women during childbearing years or retrospective views of women who had previously given birth or been offered PFMT during pregnancy. Titles and abstracts were screened for eligibility by two independent reviewers (VS, MP). Critical appraisal (using the Mixed Methods Appraisal Tool [MMAT]^21^) and data extraction (using a structured form) were conducted by two independent reviewers (VS, RJ).

**Table 1:** Electronic databases searched

MEDLINE
MEDLINE in process
Cochrane library:
HTA (Health technology assessment) database
DARE (Database of abstracts of reviews of effects)
CENTRAL (Central register of controlled trials)
EED (NHS economic evaluation database)
CDSR (Cochrane database of systematic reviews)
PROSPERO (center for reviews and dissemination register of protocols of systematic reviews)
CINAHL (Cumulative index to nursing and allied health literature)
EMBASE
PsycINFO
DoPHER (Database of promoting health effectiveness reviews)
ASSIA (Applied social sciences index and abstracts)
SSCI (Social sciences citation index)
SCI (Science citation index)
Proquest nursing

Sources were uploaded to NVivo for Teams (QSR International) and coded using a framework based on the initial research questions. Codes representing existing constructs presented across all sources were inspected and analysed by four reviewers (VS, MP, RJ, JHS), patterns and themes were identified, and new (synthetic) constructs generated. In this way, data were synthesized across sources, transformed into new explanatory themes, and developed into a synthesized argument.^19^ This method also facilitated critique, reflexivity and debate amongst the research team and our patient and public involvement (PPI) group, with whom we held a series of meetings (each lasting 2 hours) at a community center that the PPI women already attended regularly.

A full protocol for the review has been published.^22^

## 3 RESULTS

**Figure 1:**
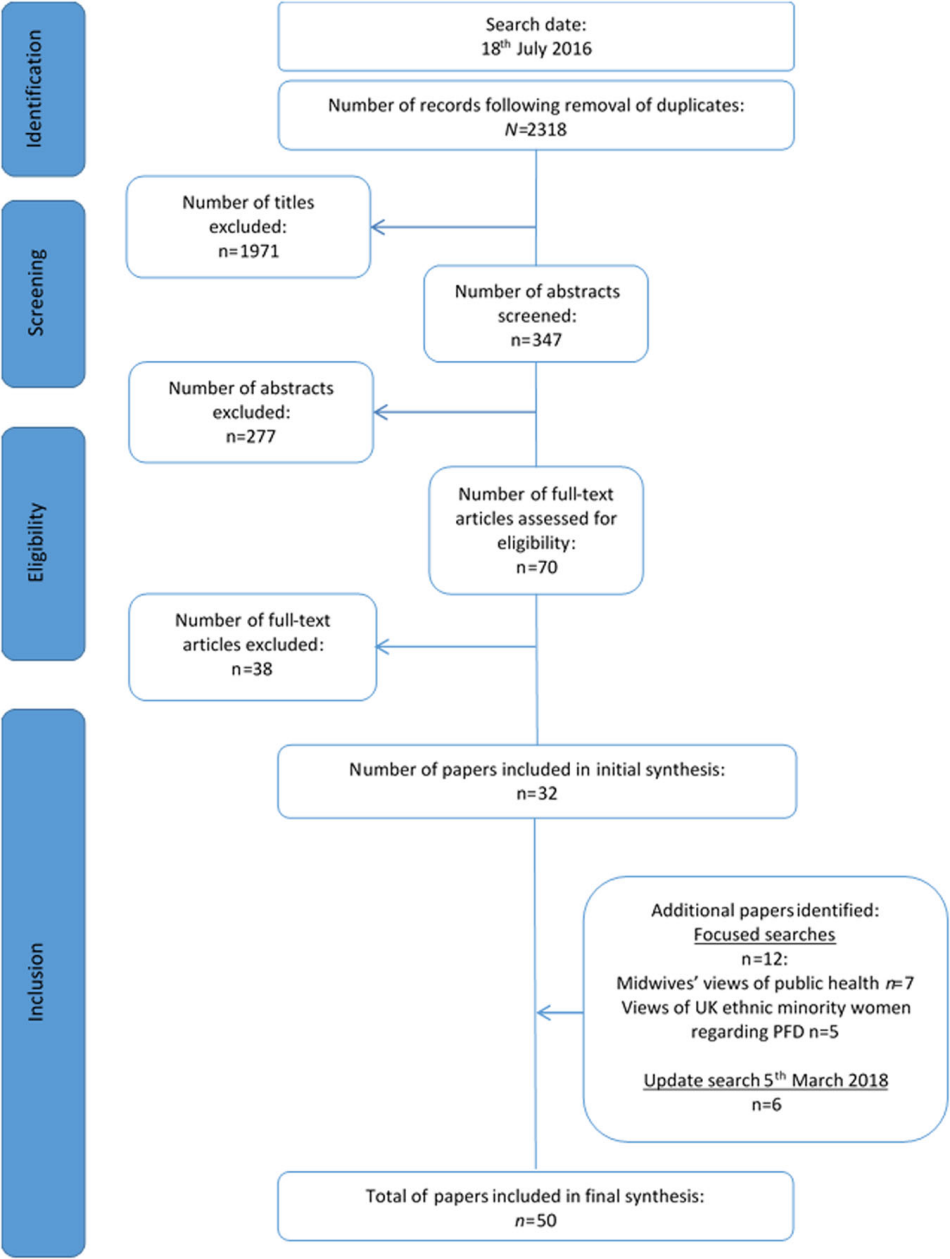
PRISMA flowchart

Figure 1 shows the flow of sources through the review. We included 50 sources in the synthesis: 5 commentaries, 2 cohort studies, 4 mixed‐method studies, 1 Q‐methodology study, 17 qualitative studies, 19 surveys, 1 implementation report, and 1 systematic review of qualitative research. Characteristics of included studies are shown in Supporting Information Materials S1. Critical appraisal of the 38 primary research studies showed four studies scored zero using the MMAT criteria,^23^ 16 scored 25%, 13 scored 50%, and five scored 75%. Key issues for quantitative studies included small sample size and no evidence of sample size calculation, minimal information about reasons for declining participation, lack of psychometric robustness for survey tools/outcome measures, and variable response rates. Reports of qualitative studies often did not consider how findings related to the context in which data were collected, or did not demonstrate reflexivity. The extent to which each study's richness contributed to the synthesis is shown in Supporting Information Materials S1.

Our synthesis identified challenges and opportunities for implementing PFMT under four themes: *Challenges and opportunities in maternity service provision for PFMT; Formation and shaping of knowledge and understanding about PFMT; Challenges and opportunities for engaging and participating in PFMT*, and; *Social and emotional challenges and opportunities*. To enhance readability, citations to sources are not included in this text but are tabulated in Supporting Information Materials S2‐5.

### 3.1 Challenges and opportunities in maternity service provision for PFMT

Routine provision of PFMT, as recommended by UK^15^ and international guidance,^13^, ^14^ is not part of antenatal services where midwives have increasing responsibilities and large workloads. Included sources did not consider the impact of competing national policies and directives, lack of professional training, or revised maternity service and funding models on PFMT implementation.

Women's uptake of PFMT may be constrained by ad hoc service provision or challenges accessing services. Women need multiple access options for PFMT, and services need to be individualized and include provision for language, literacy, and ethnic/cultural diversity.

HCPs acknowledge that limited attention is paid to pelvic floor health during pregnancy. Whilst specialist physiotherapists have traditionally been the experts in PFMT they are no longer routinely involved in UK maternity service provision, resulting in unclear professional responsibility for antenatal PFMT and poor access to specialist services.

Postnatal women would appreciate an assessment to ensure they are performing a correct PFMC. Yet there is no evidence of objective (per perineum or per vaginum) assessment of PFMC as part of antenatal or postnatal service provision, and the acceptability of antenatal objective assessment of PFMC to either women or HCPs is not known. Midwives may be opposed to undertaking objective PFMC assessment involving vaginal examination during pregnancy.^24^

Lack of guidance about how to implement antenatal PFMT, poor quality information, and a lack of continuity of care may result in organizational and individual variation in maternity service provision of PFMT. Opportunities for maternity service provision for PFMT require greater clarity around professional roles and multi‐professional working. HCPs, including midwives, and women believe that midwives are best placed to deliver antenatal continence screening and PFMT, and midwives recognize health promotion is part of their role. However, midwives' ability to implement PFMT in routine antenatal care is constrained by diminished resources, including staff shortages, lack of time in appointments, and competing priorities such as the provision of other health screening. Maternity service provision is heavily influenced by the national agenda and, as a result, midwives' clinical practice may be driven by policies or protocols rather than individual need. The resulting tick‐box approach to care may present a barrier to discussing health promotion topics when midwives face the challenge of continual role expansion. PFMT implementation requires organizational support and resources at a national and local level to make this happen in antenatal care.

### 3.2 Formation and shaping of knowledge and understanding about PFMT

Knowledge and understanding of women and HCPs may be formed and shaped by the availability and quality of information about PFMT, social and cultural norms, and attitudes and beliefs. These factors may influence the way in which individuals and groups engage in and prioritize the implementation of PFMT within their daily lives and/or clinical practice.

Women report limited knowledge about UI and PFMT, and uncertainty regarding what exercises to do and how to do them, constraining their ability to try out, adopt and maintain regular PFMT during their childbearing years. UI is framed as a postnatal issue, displacing it as priority from antenatal care for women, midwives, and organizations/maternity services alike. For example, some women believe that PFMT only needs to be done after their baby is born.

Opportunities for uptake of PFMT require improved access for women to high quality, reliable information. Culturally‐sensitive resources must be considered where literacy and language may be an additional challenge. A lack of adequate resources in other languages reduces HCPs' ability to communicate effectively with non‐English speaking women.

HCPs also express concerns regarding limited knowledge and understanding about UI and PFMT, lack of awareness of guidelines, and inadequate skills for teaching PFMT. If midwives are to implement antenatal PFMT they require additional training. Training should also explore socioeconomic, ethnic, and cultural factors to facilitate engagement with different ethnic groups.^25^

Midwives believe training opportunities are prioritized by mandatory requirements. Attending additional training may be challenging due to a lack of time and funding if PFMT is not considered a service priority. Where training for health promotion topics is available, midwives report a lack of time to embed their learning into practice due to the extensive workload pressures of an ever‐expanding midwifery role. Midwives require organizational support to attend training, and regular updates and peer support to enhance their learning.

### 3.3 Challenges and opportunities for engaging and participating in PFMT

Women and HCPs engage with and participate in PFMT to varying degrees. The extent of engagement may be influenced by other themes, such as maternity service organization and shaping of knowledge and understanding.

Included sources focus mainly on women's perspectives, mistakenly leading us to view problems with uptake of PFMT as belonging to them, in the absence of any consideration of wider system influences. The predominant narrative is that women experience several intrapersonal challenges and concerns for engaging and participating in PFMT. For example, lack of perceived importance or experience of PFMT or a lack of effectiveness from their experience of PFMT.

Lack of access to HCP‐supervised PFMT may reduce women's ability to adopt and maintain a training program. PFMC self‐efficacy is a significant predictor of PFMT behavior during pregnancy, with the potential to induce feelings of empowerment and control with PFMT. The presence of HCP‐supervised PFMT may support women to problem‐solve setbacks in engagement and participation in PFMT by using behavior change techniques such as action planning, biofeedback and prompts and cues.

HCPs experience multiple challenges to engaging in the implementation of antenatal PFMT. None of the included sources investigate how this might be enabled when maternity contacts are time‐constrained, and driven by service directives for other priorities. Insufficient time within clinical practice is a commonly cited challenge for implementing interventions, including PFMT. Competing workload pressures mean that nonmandated interventions have low priority. Lack of time to build a good relationship, particularly with young mothers, and socio‐cultural barriers including language and communication difficulties, also impacts on midwives' ability to engage women with public health topics.

Despite constraints on HCPs' ability to engage and participate in PFMT implementation, midwives feel it is their responsibility to empower women to manage their own health. Midwives recognize that implementation of health promotion messages requires dedication and commitment to facilitate consistent service provision. Included sources suggest that raising the profile of PFMT and addressing gaps in knowledge and understanding within local services and health organizations, funding bodies, professional groups and policymakers involved in maternity care may help midwives and other HCPs prioritize engagement and participation in the implementation of antenatal PFMT.

### 3.4 Social and emotional challenges and opportunities

UI is often thought of as a normal, inevitable consequence of pregnancy and childbirth, with many people viewing this condition as taboo. Women and HCPs may find it difficult to raise and talk about the topic of UI, limiting their ability to fully engage with implementing PFMT. Many women are either unaware of prevention/treatment options or do not want to bother a HCP for a problem they perceive as inevitable, minor, temporary or incurable. Further, uptake of PFMT is often difficult for women who do not easily fit a service that assumes a certain level of health literacy, capability, resources, English‐language fluency, and a “westernised” culture.

Women may feel unprepared for UI. If not asked directly, women may not raise concerns about symptoms for fear of being a burden to the health service. This sentiment was emphasized by PPI group members as a key issue for women discussing UI in busy antenatal appointments.

Midwives may have concerns about raising sensitive emotional subjects, fearing that this could negatively impact their relationship with women. Trying to discuss UI and PFMT during the first appointment (as recommended by the current NICE guidelines) may present a particular challenge. In addition, midwives believe that women may not divulge information about UI. Women in the PPI group concurred that HCPs may need to ask several times before a woman felt comfortable talking about UI and engaging in conversation about its prevention and treatment. However, midwives address other sensitive topics such as domestic abuse.

Ethnic minority women face additional social and emotional challenges. For example, maintaining cleanliness for prayer is a key concern for Muslim women, and a woman with UI may have feelings of shame and sinfulness that they do not wish to disclose to others. This may be coupled with communication challenges for some ethnic minority women, who might experience difficulty with literacy even when presented with information in their native language. Midwives need to identify the language and cultural challenges when discussing public health issues with women from different ethnic and socioeconomic groups.

Midwives working with lower socioeconomic groups also believe that cultural attitudes can be difficult to overcome. Beliefs promulgated by strong maternal influences in large, close families, may not align with the information provided by HCPs. Women may ignore HCP‐advice if other female relatives do not believe it is important or relevant. Midwives working with these client groups may be required to build relationships with and educate the whole family.

To fully engage women and encourage participation in PFMT to prevent UI there is a need to recognize social and emotional constraints around discussing these concerns including sensitivity to the social and cultural impact of UI for women from different ethnic and social backgrounds. Whilst remaining a sensitive topic, women value open and direct discussion about UI and PFMT. When led by a HCP, this can legitimize discussion about UI symptoms and foster their serious consideration by women to support engagement and participation in PFMT.

## 4 DISCUSSION

To bring together findings about the implementation of PFMT by maternity services and uptake of PFMT by women, we use the term “agency.” Agency is defined here as people's “ability to make things happen through their own actions” by interacting with other people, processes, and systems(26 p.1).

Implementation and uptake of PFMT during childbearing years requires action at the time of, or in relation to, a key phase in women's lives–namely, pregnancy. Those who need to take action include policymakers, maternity service funders and organizations, HCPs, and women themselves. The capacity of these individuals and groups to implement PFMT during childbearing years may be enhanced or diminished by the professional, organizational, and policy environment. A central mechanism of the agency is a person's belief in their ability to exercise control over their actions and environmental events (self‐efficacy).^27^ For example, inconsistent service provision may diminish women's agency if they face challenges accessing information and support for PFMT. Similarly, national directives for maternity service provision of PFMT may enhance or diminish the individual and collective agency of midwives for implementing PFMT. There are so many directives impacting on midwives and the midwifery profession that, as collective agents, they are continually required to self‐regulate and adapt to keep abreast of expanding occupational demands.^28^

National and organizational directives and resource distribution are likely to significantly influence the agency of services, HCPs, and individual women for engaging and participating in PFMT. For instance, a lack of directed resources enabling access to specialist physiotherapists may mean that agency is compromised if HCPs are unable to offer a service to women even if they want it and would use it.

Similarly, while national directives like NICE guidance could support HCP agency, for example, by allowing midwives to identify and raise this as an important area where they need additional resources and/or training, this may not be acted out in practice if resources and training are not forthcoming. Initiatives like that of the Royal College of Midwives/Chartered Society of Physiotherapy^29^, ^30^ suggest that professional bodies are taking an agentic role, that is, trying to make things happen through their actions. However, provision of an educational resource alone, in the form of an i‐learning package for midwives, places responsibility back on the individual midwife to complete the training package and does not necessarily enable collective agency at a wider professional or national policy level. In the UK, maternity plans announced at the end of 2018 include wider availability of postnatal physiotherapy that may contribute to enabling this collective agency^31^ but provides no “teeth” to address the problems in antenatal provision where there is potential for early prevention of UI and other pelvic floor disorders.

## 5 CONCLUSION

Existing research focuses on PFMT as the responsibility of individual women and HCPs, and on UI as a postnatal issue. PFMT research needs to move beyond individuals to explore and understand implementation issues at system level. Reframing of issues in line with national maternity ambitions may allow PFMT to fit more closely with a health promotion agenda, within which policymakers, organizations, HCPs, and women value the prevention of incontinence by using low‐risk, low‐cost, and proven strategies as part of women's reproductive health across the lifespan. This review shows how challenges and opportunities for implementing PFMT resonate with the priorities of the recent national maternity review, such as personalized care, continuity of care, and cross‐disciplinary, multi‐professional working.^9^, ^31^ Now is an opportune moment to revisit antenatal PFMT implementation within maternity services in the UK.

## AUTHOR CONTRIBUTIONS

MP, JH‐S, SD, EO, and CM conceptualized the study. SB designed the database search strategy with input from VS, JH‐S, MP, and RJ. VS and MP carried out screening and article selection processes. VS and RJ appraised and synthesized the included articles with critical input from MP, JH‐S, SD, HF, and EO. VS and MP wrote the first draft with critical input from JH‐S, SD, and RJ, with significant input to subsequent drafts by the remaining authors (HF, DB, RT, CD, and CM). VS and RJ facilitated the patient & public involvement groups. All authors read, reviewed and approved the final manuscript. MP is the guarantor.

## Supporting information

Supplementary information

Supplementary information

Supplementary information

Supplementary information

Supplementary information
