# Supplementary material for: Implementing pelvic floor muscle training in women's childbearing years: A critical interpretive synthesis of individual, professional, and service issues
Source: Neurourol Urodyn. 2019 Dec 17;39(2):863–70. doi: 10.1002/nau.24256 (PMC7079154; doi:10.1002/nau.24256)
Supplement: Supplementary file 1 — Supplementary information [file NAU-39-863-s001.docx]

| **Paper ID**  **(first author, publication year, country)** | **Research team characteristics** | **Stated aims/objectives** | **Participant characteristics** | | | | **Rich/ Explanatory/ Descriptive** |
| --- | --- | --- | --- | --- | --- | --- | --- |
|  |  |  | **AN** | **PN** | **HCP** | **UI/PFD** |  |
| ***Commentary/ opinion piece*** |  |  |  |  |  |  |  |
| Aston, 2009, UK (1) | Specialist physiotherapist in women’s health | Commentary on guidance for managing PFD during childbearing years, emphasising PFMT as a preventative strategy | N/A |  |  |  | N/A |
| Freeman 2013, UK (2) | Consultant in urogynaecology | Commentary on prevention of childbirth-related PFD | N/A |  |  |  | N/A |
| Mason 2001,  UK (3) | Health care research | Commentary regarding relationship between AN and PN PFME and SUI, including proposal for professional bodies to provide clear guidelines for standardised treatment and care | N/A |  |  |  | N/A |
| McClurg 2015, UK (4) | Midwifery  physiotherapy | Update on RCM and CSP 2013 initiative | N/A |  |  |  | N/A |
| Gerrard 2013, UK (5) | Midwifery  Physiotherapy | RCM/CSP initiative to prevent PFD | N/A |  |  |  | N/A |
| ***Primary research*** |  |  |  |  |  |  |  |
| ***Cohort studies*** |  |  |  |  |  |  |  |
| Fine 2007  USA (6) | Mixed academic and HP | “to describe the teaching and practicing of [PFME] before and after delivery” |  | 759 |  |  | Descriptive |
| Frawley 2014  (conference abstract)  Australia (7) | Not reported | “to trial an implementation project to increase the uptake of continence screening and [PFME] instruction, in collaboration with midwives and obstetricians” |  |  | 62 (MW, Obs) |  | Explanatory |
| ***Mixed methods studies*** |  |  |  |  |  |  |  |
| Mason 2001,  UK (8) | Academic, psychologist, midwife, registered nurse | Aimed to investigate Instruction in pelvic floor exercises provided to women during pregnancy or following delivery:  a) the instruction provided routinely to women during the AN and PN period  b) whether the service met recommendations in literature  c) needs of the women |  | 572 |  |  | Descriptive |
| McNeill, 2012^a^ (9) | Female authors, characteristics and affiliations not reported | “To identify the scope of current midwifery pre-registration educational provision in relation to public health and to explore the perspectives of midwives and midwifery students about the public health role of the midwife”. Included survey of 29 Higher Education Institutions. Focus group participants included midwifery students, registered midwives, managers, public health specialists and educationalists. |  |  | 59 |  | Explanatory |
| Wells, 2007^b^ (10) | Female Nurse Consultant at an Integrated bladder and Bowel Care,; male Senior Lecturer and Consultant in Geriatric Medicine | “To evaluate the needs and requirements for continence care provision within the Bangladeshi community in Camden and Islington by employing a healthcare advocate to carry out a time limited project.” |  |  | 10 | 129^c^ | Explanatory |
| Xing 2017^d^ (11)  China | Lecturer, Registered Nurse, Deputy Nurse Director | “To promote PFMT to prevent UI among antenatal and postnatal women in an obstetric and gynaecological hospital in China.” | 30 |  | 18 (nurses) |  | Explanatory |
| ***Q methodology*** |  |  |  |  |  |  |  |
| Herron-Marx 2007  UK (12) | Midwifery  psychology  nursing | “To critically examine women’s experiences of enduring perineal morbidity” |  | 20 |  | PFD | Explanatory |
| ***Qualitative studies*** |  |  |  |  |  |  |  |
| Ashworth, 1993, UK (13) | Sociology  Psychology | To understand “social and emotional consequences of non-compliance with [PFME]” |  | 28* |  | UI | Explanatory |
| Buurman 2013  Netherlands (14) | GPs, female, academics | “to improve our understanding of women’s cognitions relating to pelvic ﬂoor dysfunction and of impediments to help-seeking behaviour so as to improve the match between patients’ needs and medical help” |  | 26 |  |  | Explanatory |
| Chaliha, 1999^b^ (15) | Not known | “To explore religious and cultural practices within ethnic minorities that may effect [*sic*] the experience and ideas sufferers have of incontinence” (34 women interviewed: 7 Muslims, 9 Hindus, 6 Jewish, 3 Buddhists and 9 Christians) |  |  |  | 34^c^ | Explanatory |
| Chiarelli 1999  Australia (16) | Female; Lead author - physiotherapy | “1. To examine women's knowledge and beliefs about UI its prevention and its progressive nature; 2. To explore women's perceived susceptibility to UI; 3. To explore women's levels of perceived severity of UI; 4. To explore the attitudes of women to their experiences of UI; 5. To examine women's knowledge and beliefs about pelvic floor exercises; 6. To explore women's beliefs about their ability to perform a PFM contraction; 7. To ascertain if women have any experience with or ideas about [PFME] compliance aiding strategies; 8. To discover which health care professional the women might see as a highly credible source of information about UI and [PFME]; 9. To explore other data emerging from the focus groups that might be relevant to continence promotion.” |  | 37 |  |  | Explanatory |
| Cooper 2015  UK (17) | Physiotherapy | “to investigate the factors that inﬂuence primigravid women undergoing routine antenatal care to adhere to PFMEs” | 4 |  |  |  | Explanatory |
| Doi, 2014^a^ (18) | Male and female authors, university based school of public health research, professional qualifications not reported | Exploration of “midwives’ attitudes and practices regarding alcohol screening and [alcohol brief interventions] in order to understand why they are relatively underutilized in antenatal care settings compared to other clinical settings” |  |  | 21 |  | Explanatory |
| Doshani, 2007^b^ (19) | 2 female, 2 male authors, based at clinical and academic institutions. Bilingual author facilitated focus groups | “To explore the views and experiences of women in the South Asian Indian community in Leicester about urinary incontinence and their perceptions of available care” |  |  |  | 24^c^ | Explanatory |
| Gillard 2010  UK (20) | Physiotherapy and academic | “to determine the factors that motivated women to do [PFMEs] following a perineal tear at delivery within the conceptual framework of the [Health Belief Model] |  | 10 |  |  | Explanatory |
| Herberts, 2012^a^ (21) | Female authors, chartered psychologists and senior lecturer | “To identify and juxtapose midwives’ perceptions of providing stop-smoking advice and pregnant smokers’ perceptions of stop-smoking services” | 10 |  | 15 |  | Explanatory |
| Heslehurst, 2013^a^ (22) | Female authors  Academics and clinicians (midwife and public health consultant) | “To explore midwives’ perceptions of training and education requirements in relation to maternal obesity” |  |  | 46 |  | Explanatory |
| Lee, 2012^a^ (23) | Female authors including Project manager in Public Health Clinical Effectiveness Unit, NHS trust; Public Health Analyst, National Institute for Health and Clinical Excellence; Consultant midwife in public health, NHS | To explore “midwives’ opinions and working practice on health promotion” |  |  | 15 |  | Explanatory |
| Mason 2001, UK (24) | Academic, psychologist, midwife, registered nurse | ”to consider whether women who experience stress incontinence following childbirth are provided with information on the condition, whether they seek help and what help they receive” |  | 42 |  | UI | Descriptive |
| Mason 2001, UK (25) | Academic, psychologist, midwife, registered nurse | “To determine whether women practice PFME following routine instruction from HPs, during pregnancy or post-delivery. Motivation to exercise or not was also investigated” |  | 42 |  | UI | Descriptive |
| Sanders, 2016^a^ (Hunter, 2015)^a^ (26, 27) | Female authors, registered midwives & midwifery academics | “To identify student midwives’, midwives’ and midwifery support works’ current knowledge of and involvement in the public health agenda in England” |  |  | 60 |  | Explanatory |
| Sange, 2008^b^ (28) | Female and male authors. Academics and clinicians with backgrounds in nursing and gynaecology. Lead researcher from an Asian background, able to speak Urdu. | “To explore the religious and cultural inﬂuences on help-seeking behaviour and decision-making in South Asian Muslim women with UI.” |  |  |  | 9^c^ | Explanatory |
| Wagg 2017^d^ (29)  UK | Female  Nurse, Professor of Nursing, Reader in evidence based health care | “To explore, describe and enhance understanding of women’s experiences, beliefs and knowledge of urinary symptoms in the postpartum period and also sought to understand the perceptions of health professionals of these issues.” |  | 15 | 10  (mixed) |  | Explanatory |
| Wilkinson, 2001^b^ (30) | Female registered nurse and Clinical Leader, Continence Care | “To explore [Pakistani women’s] perceptions and experiences of urinary incontinence.” |  |  |  | 6^c^ | Explanatory |
| ***Surveys*** |  |  |  |  |  |  |  |
| Bo 2007, Norway (31) | Physiotherapy | “To estimate the association between PFMT and several demographic and health-related factors that may influence PFMT 6 months’ postpartum” |  | 17774 |  |  | Explanatory |
| Butterfield 2007  Australia (32) | Not stated (Maternity services) | “To obtain knowledge and information on midwives assessment and management practices of UI in childbearing women and to explore midwives risk factors associated with development of UI”. |  |  | 225 (MW) |  | Descriptive |
| Chiarelli 1997  Australia (33) | Consultant physiotherapist Continence adviser and evaluation officer | To examine: “Prevalence of incontinence during pregnancy; whether specific variables were associated with continence status, the extent to which women reported receiving continence advice from healthcare professionals during pregnancy, the extent to which women reported PFM assessment during vaginal examinations; women’s attitudes to provision of continence advice and assessment of PFM strength during pregnancy” |  | 304 |  |  | Descriptive |
| Chiarelli 2003  Australia (34) | Female, physiotherapy | To explore the acceptability of a urinary continence promotion programme for postpartum women |  | 348 |  |  | Descriptive |
| Cooke 2017^d^ (35)  International | Academic, Obs and Gynae | “To determine urogynaecology providers’ knowledge of risk and protective factors for postnatal PFD, and to assess their practice patterns in postnatal PFD counselling.” |  |  | 372 (mixed) |  | Descriptive |
| Dessie 2015  USA (36) | Medical doctor, doctor of science, master of public health | “To assess prenatal counselling practices of obstetrical providers related to postpartum [PFD]” |  |  | 173 (Obs) |  | Descriptive |
| Geynisman-Tan 2017^d^ (37)  USA | Medical doctors | “to describe the knowledge on pelvic floor disorders among a cross section of pregnant women.” | 402 |  |  |  | Descriptive |
| Guerrero 2007  UK (38) | Urogynaecology service (lead author) | “to discover how often women perform [PFME] in the antenatal period and how they wished to be taught” | 54 |  | 75 (mixed) |  | Descriptive |
| Hermansen 2010  Denmark (39) | Nursing  Obs and Gynae, Academic nursing | “To determine where and from whom postpartum women recalled receiving information about [UI] and [PFMEs], the helpfulness of this information, and their preferred sources of help with UI.” |  | 266 |  |  | Descriptive |
| Hill 2017^d^ (40)  Australia | Physiotherapy  Academic | “To evaluate pregnant women’s levels of awareness, knowledge, and beliefs about the pelvic floor muscles (PFMs) and PFMEs.” | 633 |  |  |  | Descriptive |
| Ismail 2009  UK (41) | Urogynaecology and PF Unit. | “to assess patient awareness of and practice of PFME in first pregnancy” and to look at sources of information and ways to improve patient compliance |  | 223 |  |  | Descriptive |
| Logan 2001  UK (42) | Nursing  Continence services manager | “to improve local practice in the provision of information on urinary stress in continence by investigating whether [PFMEs] are taught during pregnancy and whether women understand the importance of regular [PFMEs]” |  | 99 | 143 (MW) |  | Descriptive |
| Mason 2001,  UK (43) | Academic, psychologist, midwife, registered nurse | “to investigate any link between antenatal PFME performed in the ‘real world’ and reported symptoms of post-partum stress incontinence” | 717 | 572 |  |  | Descriptive |
| Moossdorff-Steinhauser 2015  Netherlands (44) | Based at pelvic care centre of obs and gyn of Maastricht university medical centre/atrium medical centre Pakstad, Netherlands | “to analyse willingness to participate in an intensive preventative pelvic floor muscle training program and influencing factors, from the perspective of postpartum women, for participation” |  | 169 |  |  | Descriptive |
| Neels 2016^d^ (45)  Belgium | Academic, physiotherapy, obs & gynae | “To investigate the level of knowledge and information on pelvic floor dysfunction in peripartum and menopausal women” | 295 | 107 |  |  | Descriptive |
| Sacomori 2010  Brazil (46) | Not reported | “to examine the relationship between pelvic floor muscle strength and body self-perception variables in pregnant women and determine the influence of parity on the strength of contraction of the pelvic floor muscles and on body self-perception of pregnant women” | 35 |  |  |  | Explanatory |
| Whitford 2007 (47)  UK (linked to 2011 publication) | Academic/  midwifery | “To find out the number of pregnant women who had access to information about [PFME] and whether these women reported the practice of [PFME] during pregnancy. To establish the prevalence of stress UI in these women" Aimed to interview 200 women | 289 |  |  |  | Explanatory |
| Whitford 2011  UK (48) | None supplied; school of nursing and midwifery | “To investigate the motivation of pregnant women towards the practice of [PFME] during pregnancy using the [Revised Theory of Planned Behaviour], including past behaviour as an additional element to improve the explanatory power of the model.” | 289 | 163 |  |  | Explanatory |
| Wilson 2014  Australia (49) | Physiotherapy (1^st^ author)  No other info provided about other authors | To identify the scope of [AN education] classes in [Western Australia] [and] what information was provided to women about [PFMEs] |  |  | 26 (PT) |  | Descriptive |
| ***Non-primary research*** |  |  |  |  |  |  |  |
| ***Systematic review*** |  |  |  |  |  |  |  |
| Hay-Smith 2015 (50) | Behavioural psychology, Physiotherapy, international representation from NZ, UK, USA, Canada, Australia | “To locate and summarize the ﬁndings of qualitative studies exploring the experience of and adherence to [PFMT] to recommend future directions for practice and research.” | N/A |  |  |  | N/A |

*AN=antenatal; CSP=Chartered Society of Physiotherapy; GP=general practitioner; HCP=healthcare professional; ID=identification; MW=midwife; N/A=not applicable; Obs=obstetrician; PFD=pelvic floor dysfunction; PFME=pelvic floor muscle exercise; PFMT=pelvic floor muscle training; PN=postnatal; RCM=Royal College of Midwives; SUI=stress urinary incontinence; UI=urinary incontinence; *=study participants were mothers aged 25 to 55 years old; [ ]=abbreviation or full text inserted by review authors; ^a^ located through purposive search of views of midwives regarding their health promotion role; ^b^ located through purposive search of views of UK ethnic minority women regarding PFD; ^c^ focus of research was on exploring perceptions and understandings in communities - number of participants with or without continence problems not reported; ^d^studies identified in updated search. Data from these studies have not been added to the data tables as they did not provide any additional information or alter any conclusions from those included in original searches*

References

1. Aston B. Preventing pelvic floor dysfunction: childbearing women deserve better care. Journal of Family Health Care. 2009;19(5):150-1.

2. Freeman RM. Can we prevent childbirth-related pelvic floor dysfunction? BJOG: An International Journal of Obstetrics & Gynaecology. 2013;120(2):137-40.

3. Mason L. Evidence-based midwifery in action Guidelines on the teaching of pelvic floor exercises. British Journal of Midwifery. 2001;9(10).

4. McClurg D, Gerrard J, Ten Hove R. Reducing the incidence of incontinence. British Journal of Midwifery. 2015;23(1):17-20.

5. Gerrard J, ten Hove R. RCM/CSP Joint Statement on Pelvic Floor Muscle Exercise: Improving outcomes for women following pregnancy and birth. London: Royal College of Midwives and Chartered Society of Physiotherapy; 2013.

6. Fine P, Burgio K, Borello-France D, Richter H, Whitehead W, Weber A, et al. Teaching and practicing of pelvic floor muscle exercises in primiparous women during pregnancy and the postpartum period. American Journal of Obstetrics and Gynecology. 2007;197(1):107.e1-.e5.

7. Frawley H, Chiarelli P, Gunn J. Uptake of antepartum continence screening and pelvic floor muscle exercise instruction by maternity care providers: An implementation project. Neurourology and Urodynamics. 2014;33 (6):976-7.

8. Mason L, Glenn S, Walton I, Hughes C. The instruction in pelvic floor exercises provided to women during pregnancy or following delivery. Midwifery. 2001;17(1):55-64.

9. McNeill J, Doran J, Lynn F, Anderson G, Alderdice F. Public health education for midwives and midwifery students: a mixed methods study. BMC Pregnancy & Childbirth. 2012;12:142.

10. Wells M, Wagg A. Integrated continence services and the female Bangladeshi population. British Journal of Nursing. 2007;16(9):516-9.

11. Xing W, Zhang Y, Gu C, Lizarondo L. Pelvic floor muscle training for the prevention of urinary incontinence in antenatal and postnatal women: a best practice implementation project. JBI Database of Systematic Reviews and Implementation Reports. 2017;15(2):567-83.

12. Herron-Marx S, Williams A, Hicks C. A Q methodology study of women's experience of enduring postnatal perineal and pelvic floor morbidity. Midwifery. 2007;23(3).

13. Ashworth PD, Hagan MT. Some social consequences of non-compliance with pelvic floor exercises. Physiotherapy. 1993;79(7):465-71.

14. Buurman MBR, Lagro-Janssen ALM. Women's perception of postpartum pelvic floor dysfunction and their help-seeking behaviour: a qualitative interview study. Scandinavian Journal of Caring Sciences. 2013;27(2):406-13.

15. Chaliha C, Stanton SL. The ethnic cultural and social aspects of incontinence - A pilot study. International Urogynecology Journal. 1999;10(3):166-70.

16. Chiarelli P, Cockburn J. The development of a physiotherapy continence promotion program using a customer focus. Australian Journal of Physiotherapy. 1999;45(2):111-9.

17. Cooper H, Carus C. Factors affecting women’s adherence with pelvic floor muscle exercises in a first pregnancy: a qualitative interview study. 2015.

18. Doi L, Cheyne H, Jepson R. Alcohol brief interventions in Scottish antenatal care: a qualitative study of midwives' attitudes and practices. BMC Pregnancy & Childbirth. 2014;14:170.

19. Doshani A, Pitchforth E, Mayne CJ, Tincello DG. Culturally sensitive continence care: a qualitative study among South Asian Indian women in Leicester. Family Practice. 2007;24(6):585-93.

20. Gillard S, Shamley D. Factors motivating women to commence and adhere to pelvic floor muscle exercises following a perineal tear at delivery: the influence of experience. Journal of the Association of Chartered Physiotherapists in Women's Health. 2010.

21. Herberts C, Sykes C. Midwives' perceptions of providing stop-smoking advice and pregnant smokers' perceptions of stop-smoking services within the same deprived area of London. Journal of Midwifery & Women's Health. 2012;57(1):67-73.

22. Heslehurst N, Russell S, McCormack S, Sedgewick G, Bell R, Rankin J. Midwives perspectives of their training and education requirements in maternal obesity: a qualitative study. Midwifery. 2013;29(7):736-44.

23. Lee DJ, Haynes CL, Garrod D. Exploring the midwife's role in health promotion practice. British Journal of Midwifery. 2012;20(3).

24. Mason L, Glenn S, Walton I, Hughes C. Women's reluctance to seek help for stress incontinence during pregnancy and following childbirth. Midwifery. 2001;17(3):212-21.

25. Mason L, Glenn S, Walton I, Hughes C. Do women practise pelvic floor exercises during pregnancy or following delivery? Physiotherapy. 2001;87(12):662-70.

26. Hunter B, Sanders J, Warren L. Exploring the Public Health Role of Midwives and Maternity Support Workers: Final Report. Cardiff: Cardiff University; 2015 25 February 2015.

27. Sanders J, Hunter B, Warren L. A wall of information? Exploring the public health component of maternity care in England. Midwifery. 2016;34:253-60.

28. Sange C, Thomas L, Lyons C, Hill S. Urinary incontinence in Muslim women. Nursing Times. 2008;104(25):49-52.

29. Wagg AR, Kendall S, Bunn F. Women’s experiences, beliefs and knowledge of urinary symptoms in the postpartum period and the perceptions of health professionals: a grounded theory study. Primary Health Care Research & Development. 2017;18:448-62.

30. Wilkinson K. Pakistani women's perceptions and experiences of incontinence. Nursing Standard. 2001;16(5):33-9.

31. Bø K, Owe KM, Nystad W. Which women do pelvic floor muscle exercises six months' postpartum? American Journal of Obstetrics & Gynecology. 2007;197(1).

32. Butterfield YC, O’Connell B, Phillips D. Peripartum urinary incontinence: A study of midwives’ knowledge and practices. Women and Birth. 2007;20(2):65-9.

33. Chiarelli P, Campbell E. Incontinence during pregnancy. Prevalence and opportunities for continence promotion. Australian & New Zealand Journal of Obstetrics & Gynaecology. 1997;37(1):66-73.

34. Chiarelli P, Murphy B, Cockburn J. Acceptability of a urinary continence promotion programme to women in postpartum. BJOG: An International Journal of Obstetrics & Gynaecology. 2003;110(2):188-96.

35. Cooke CM, O'Sullivan OE, O'Reilly BA. Urogynaecology providers' attitudes towards postnatal pelvic floor dysfunction. Int Urogynecol J. 2018;29(5):751-66.

36. Dessie SG, Hacker MR, Dodge LE, Elkadry EA. Do Obstetrical Providers, Counsel Women About Postpartum Pelvic Floor Dysfunction? Journal of Reproductive Medicine. 2015;60(5-6):205-10.

37. Geynisman-Tan JM, Taubel D, Asfaw TS. Is Something Missing From Antenatal Education? A Survey of Pregnant Women's Knowledge of Pelvic Floor Disorders. Female Pelvic Medicine & Reconstructive Surgery. 2018;24(6):440-3.

38. Guerrero K, Owen L, Hirst G, Emery S. Antenatal pelvic floor exercises: A survey of both patients' and health professionals' beliefs and practice. Journal of Obstetrics and Gynaecology. 2007;27(7):684-7.

39. Hermansen IL, O'Connell B, Gaskin CJ. Are postpartum women in denmark being given helpful information about urinary incontinence and pelvic floor exercises? Journal of Midwifery & Women's Health. 2010;55(2):171-4.

40. Hill A-M, McPhail SM, Wilson JM, Berlach RG. Pregnant women’s awareness, knowledge and beliefs about pelvic floor muscles: a cross-sectional survey. Int Urogynecol J. 2017;28:1557-65.

41. Ismail SI. An audit of NICE guidelines on antenatal pelvic floor exercises. International Urogynecology Journal. 2009;20(12):1417-22.

42. Logan K. Audit of advice provided on pelvic floor exercises. Professional Nurse. 2001;16(9).

43. Mason L, Glenn S, Walton I, Hughes C. The relationship between between ante-natal pelvic floor muscle exercises and post-partum stress incontinence. Physiotherapy. 2001;87(12):651-3.

44. Moossdorff-Steinhauser HFA, Albers-Heitner P, Weemhoff M, Spaanderman MEA, Nieman FHM, Berghmans B. Factors influencing postpartum women's willingness to participate in a preventive pelvic floor muscle training program: A web-based survey. European Journal of Obstetrics Gynecology and Reproductive Biology. 2015;195:182-7.

45. Neels H, Tjalma WAA, Wyndaele J-J, de Wachter S, Wyndaele M, Vermandel A. Knowledge of the pelvic floor in menopausal women and in peripartum women. J Phys Ther Sci. 2016;28:3020-9.

46. Sacomori C, Cardoso FL, Vanderlinde C. Pelvic floor muscle strength and body self-perception among Brazilian pregnant women. Physiotherapy. 2010;96(4):337-43.

47. Whitford HM, Alder B, Jones M. A cross-sectional study of knowledge and practice of pelvic floor exercises during pregnancy and associated symptoms of stress urinary incontinence in North-East Scotland. Midwifery. 2007;23(2):204-17.

48. Whitford HM, Jones M. An exploration of the motivation of pregnant women to perform pelvic floor exercises using the revised theory of planned behaviour. British Journal of Health Psychology. 2011;16(4):761-78.

49. Wilson J, Berlach RG, Hill A-M. An audit of antenatal education facilitated by physiotherapists in Western Australian public hospitals. Australian & New Zealand Continence Journal. 2014;20(2).

50. Hay‐Smith J, Dean S, Burgio K, McClurg D, Frawley H, Dumoulin C. Pelvic‐floor‐muscle‐training adherence “modifiers”: A review of primary qualitative studies—2011 ICS State‐of‐the‐Science Seminar research paper III of IV. Neurourology and Urodynamics. 2015;34(7):622-31.
