# Supplementary material for: Implementing pelvic floor muscle training in women's childbearing years: A critical interpretive synthesis of individual, professional, and service issues
Source: Neurourol Urodyn. 2019 Dec 17;39(2):863–70. doi: 10.1002/nau.24256 (PMC7079154; doi:10.1002/nau.24256)
Supplement: Supplementary file 2 — Supplementary information [file NAU-39-863-s002.docx]

**Challenges and opportunities in maternity service provision for PFMT**

| **Perspective (area)** | **Synthetic constructs: Challenges/concerns** | **Data source** | **Synthetic constructs: Opportunities** | **Data source** |
| --- | --- | --- | --- | --- |
| **Women (education)** | Routine recommendation, or education about PFME is ad hoc  Minimal or no advice/instruction/supervision for PFME during or after pregnancy  Quality of information provided is highly variable  Feel unprepared for PFD by HCP  HCPs may assume knowledge, for example, in women who have already had children | (1-14) | Midwives are best placed to teach PFMT  PFMT instruction should be supervised  Women would like leaflets/written information to be accompanied by verbal information and explanation/instruction with opportunity to ask questions | (1, 6, 15-18) |
|  | Poor timing of PFME/PFMT information delivery - often received postnatally e.g. leaflet on post-natal ward is too late and inappropriate timing | (4, 9, 11, 15, 16) | More support from HCPs to promote pelvic floor health during pregnancy, rather than in PN period  Access to peer support for PFMT would be helpful | (1, 6, 8, 9, 15, 19) |
| **Women (assessment)** | PFMC is not assessed | (4-6, 9, 14, 18) | Minimal embarrassment for vaginal examination of PFMC in postnatal period  Self-assessment of PFMC acceptable in postnatal period | (4, 5, 9, 18) |
| **Women (access to services)** | Challenges accessing services that provide PFME education/PFMT due to:   - time of day - costs - travel distance/time - transport - mode of delivery - antenatal classes not attended by all women - age - younger women less likely to have information - cultural variation – ethnic minority women taught about PFME less often and learn less from other sources - socio-economic status – more educated and more affluent women more likely to access information | (6-9, 12, 18, 20) | AN classes may provide opportunity for supervised PFMT instruction, but variable preference for individual versus class-based delivery  Target women who are less likely to access information, e.g. younger women, those from deprived backgrounds, women in their first pregnancy | (5, 9, 10, 12, 13, 15, 16, 21) |
|  | Minimal contact with specialist services for PFD/PFMT | (8, 12) | Provide access to specialist advice for women with PFD/UI symptoms | (8, 12) |
|  | Limited access to culturally specific services or resources for ethnically diverse communities | (6, 19, 22) | Offer culturally specific resources, e.g. via talk-based media | (19, 23) |
| **HCPs (organisation of services)** | Unclear professional responsibility for teaching PFMT in pregnancy  Limited input of specialist HCPs to routine antenatal care (e.g. specialist physiotherapists)  Lack of awareness of/referral to specialist services/multi-disciplinary team  Difficulty communicating with specialist services | (1, 12, 20, 24-30) | Midwives are well placed to deliver AN PFMT/health promotion interventions  Midwives’ passion, dedication and commitment to provide best possible care can facilitate delivery of public health messages  Refer ‘high risk’ women to specialist services, e.g. intensive PFMT with physiotherapist/continence advisor | (1, 2, 9, 12, 21, 25, 26, 28-35) |
|  | Inconsistent provision of services for PFME/PFD, e.g. limited attention to PFM health, AN continence screening not routine, limited advice giving regarding PFME/PFD, variable instruction for PFMT, variable quality of instruction | (1, 2, 36, 37) | Offer routine continence screening in AN clinics to identify ‘at risk’ groups and pelvic floor health promotion at each AN appointment/every opportunity, including routine recommendation for PFMT, opportunity for discussion and timely treatment | (1, 4, 25, 26, 38) |
|  | Poor timing of information delivery - e.g. giving information before discharge from hospital is not effective | (2, 34, 39) | Offer PFD/UI advice and supervised PFMT instruction during pregnancy, and reinforce in postnatal period | (1, 2, 4, 10, 13, 15, 33, 36) |
|  | Unable to offer continuity of care | (34) | Requires review of time pressures, workload and staff resources to allow continuity of care | (38) |
| **HCPs (education)** | Limited awareness/adherence to guidelines for teaching PFME/PFMT | (20, 37) | Raise awareness of guidelines that recommend women should be offered supervised PFMT in their first pregnancy  Agreement from professional bodies to support implementation of PFMT by midwives | (25-28) |
| **HCPs (assessment)** | Midwifery opposition to assessment of PFMC | (37) | Identify acceptable method for assessing correct PFMC | (4, 9, 11, 18, 21, 27, 37, 40) |
|  | Need for female HCP for some ethnic minority women may present a challenge | (19) | *No data relating to opportunities for HCPs for this construct* |  |
| **HCPs (public health approach)** | Midwives describe providing public health education (providing verbal and/or written information and signposting to services), rather than health promotion (personalised support to enable women to lead healthier lives) | (35) | Focus on mothers’ health to improve prevention and treatment of PFD related to pregnancy and childbirth | (3) |
| **Service/ Organisation/ Policy** | Unclear guidelines or recommendations for PFMT/UI during pregnancy/childbearing years  Lack of guidance or policy from professional bodies  Limited resources available for provision of PFMT in maternity services, e.g. time, staff, training/educational resources | (2, 13, 15, 24, 27)  (25) | Raise awareness of guidelines that recommend women should be offered supervised PFMT in their first pregnancy  Agreement from professional bodies to support implementation of PFMT by midwives | (25-28) |
|  | Organisational variation in quality and detail of PFMT information provision | (2, 17, 20) | Revise healthcare policy to ensure PFD/UI is discussed with all women during/after pregnancy  Make instruction in PFMT explicit in AN service policy/protocols | (8, 9, 13) |
|  | *No data related to organisational challenges/concerns for this construct* |  | Consider suitable mode of delivery for PFMT:   - PFMT with supervision more effective for preventing PFD than without - Individual versus group supervised PFMT - AN classes - Web-based education - Written information and instruction - Culturally specific talk-based media - Brief interventions for delivery in routine AN care (5-10mins) | (1, 9, 12, 13, 15, 17, 19, 20, 23, 33, 37) |
|  | Lack of responsibility for multi-disciplinary working Challenges accessing specialist services | (24, 29, 30) | Provide organisational support and access to resources for women and service providers to facilitate pelvic floor health promotion in AN care:   - adequate time and continuity to implement consistent UI advice and PFMT instruction - access to specialist services for UI (physiotherapy/continence services) - high quality educational materials | (2, 25, 27, 28, 32, 36, 38) |
|  | Influence of national agenda on service priorities  Public health agenda driven by policy/protocol rather than individual need  ‘Tick box’ approach to care may be a barrier to discussing public health topics in clinical practice | (29, 30, 33, 34) | Provide clear guidance on professional and inter-professional responsibility for PFMT in AN care   - Midwives are best placed to teach PFMT with multi-disciplinary support - Make better use of maternity support workers - Improve multi-disciplinary working to provide coordinated approach to preventing UI and teaching effective PFMT - Develop clear service standards and transparent local care pathways for PFD including referral for specialist continence services as required | (1, 2, 5, 8, 9, 12, 13, 18, 20, 21, 24-28, 30-32) |

*AN=antenatal; HCP=healthcare professional; PFD=pelvic floor dysfunction; PFM=pelvic floor muscle; PFMC=pelvic floor muscle contraction; PFME=pelvic floor muscle exercise; PFMT=pelvic floor muscle training; UI=urinary incontinence*

References

1. Guerrero K, Owen L, Hirst G, Emery S. Antenatal pelvic floor exercises: A survey of both patients' and health professionals' beliefs and practice. Journal of Obstetrics and Gynaecology. 2007;27(7):684-7.

2. Logan K. Audit of advice provided on pelvic floor exercises. Professional Nurse. 2001;16(9).

3. Buurman MBR, Lagro-Janssen ALM. Women's perception of postpartum pelvic floor dysfunction and their help-seeking behaviour: a qualitative interview study. Scandinavian Journal of Caring Sciences. 2013;27(2):406-13.

4. Chiarelli P, Campbell E. Incontinence during pregnancy. Prevalence and opportunities for continence promotion. Australian & New Zealand Journal of Obstetrics & Gynaecology. 1997;37(1):66-73.

5. Chiarelli P, Cockburn J. The development of a physiotherapy continence promotion program using a customer focus. Australian Journal of Physiotherapy. 1999;45(2):111-9.

6. Fine P, Burgio K, Borello-France D, Richter H, Whitehead W, Weber A, et al. Teaching and practicing of pelvic floor muscle exercises in primiparous women during pregnancy and the postpartum period. American Journal of Obstetrics and Gynecology. 2007;197(1):107.e1-.e5.

7. Hay-Smith EJC, Dean S, Burgio K, McClurg D, Frawley H, Dumoulin C. Pelvic floor muscle training adherence 'modifiers': A review of primary qualitative studies. ICS State of the Science Seminar research paper II of IV. Neurourology and Urodynamics. 2015;34(7):622-31.

8. Herron-Marx S, Williams A, Hicks C. A Q methodology study of women's experience of enduring postnatal perineal and pelvic floor morbidity. Midwifery. 2007;23(3).

9. Mason L, Glenn S, Walton I, Hughes C. The instruction in pelvic floor exercises provided to women during pregnancy or following delivery. Midwifery. 2001;17(1):55-64.

10. Mason L, Glenn S, Walton I, Hughes C. Women's reluctance to seek help for stress incontinence during pregnancy and following childbirth. Midwifery. 2001;17(3):212-21.

11. Mason L, Glenn S, Walton I, Hughes C. Do women practise pelvic floor exercises during pregnancy or following delivery? Physiotherapy. 2001;87(12):662-70.

12. Whitford HM, Alder B, Jones M. A cross-sectional study of knowledge and practice of pelvic floor exercises during pregnancy and associated symptoms of stress urinary incontinence in North-East Scotland. Midwifery. 2007;23(2):204-17.

13. Hermansen IL, O'Connell B, Gaskin CJ. Are postpartum women in denmark being given helpful information about urinary incontinence and pelvic floor exercises? Journal of Midwifery & Women's Health. 2010;55(2):171-4.

14. Cooper H, Carus C. Factors affecting women’s adherence with pelvic floor muscle exercises in a first pregnancy: a qualitative interview study. 2015.

15. Moossdorff-Steinhauser HFA, Albers-Heitner P, Weemhoff M, Spaanderman MEA, Nieman FHM, Berghmans B. Factors influencing postpartum women's willingness to participate in a preventive pelvic floor muscle training program: A web-based survey. European Journal of Obstetrics Gynecology and Reproductive Biology. 2015;195:182-7.

16. Ashworth PD, Hagan MT. Some social consequences of non-compliance with pelvic floor exercises. Physiotherapy. 1993;79(7):465-71.

17. Bø K, Owe KM, Nystad W. Which women do pelvic floor muscle exercises six months' postpartum? American Journal of Obstetrics & Gynecology. 2007;197(1).

18. Chiarelli P, Murphy B, Cockburn J. Acceptability of a urinary continence promotion programme to women in postpartum. BJOG: An International Journal of Obstetrics & Gynaecology. 2003;110(2):188-96.

19. Wells M, Wagg A. Integrated continence services and the female Bangladeshi population. British Journal of Nursing. 2007;16(9):516-9.

20. Wilson J, Berlach RG, Hill A-M. An audit of antenatal education facilitated by physiotherapists in Western Australian public hospitals. Australian & New Zealand Continence Journal. 2014;20(2).

21. Ismail SI. An audit of NICE guidelines on antenatal pelvic floor exercises. International Urogynecology Journal. 2009;20(12):1417-22.

22. Wilkinson K. Pakistani women's perceptions and experiences of incontinence. Nursing Standard. 2001;16(5):33-9.

23. Doshani A, Pitchforth E, Mayne CJ, Tincello DG. Culturally sensitive continence care: a qualitative study among South Asian Indian women in Leicester. Family Practice. 2007;24(6):585-93.

24. Mason L. Evidence-based midwifery in action Guidelines on the teaching of pelvic floor exercises. British Journal of Midwifery. 2001;9(10).

25. McClurg D, Gerrard J, Ten Hove R. Reducing the incidence of incontinence. British Journal of Midwifery. 2015;23(1):17-20.

26. Freeman RM. Can we prevent childbirth-related pelvic floor dysfunction? BJOG: An International Journal of Obstetrics & Gynaecology. 2013;120(2):137-40.

27. Aston B. Preventing pelvic floor dysfunction: childbearing women deserve better care. Journal of Family Health Care. 2009;19(5):150-1.

28. Gerrard J, ten Hove R. RCM/CSP Joint Statement on Pelvic Floor Muscle Exercise: Improving outcomes for women following pregnancy and birth. London: Royal College of Midwives and Chartered Society of Physiotherapy, 2013.

29. Heslehurst N, Russell S, McCormack S, Sedgewick G, Bell R, Rankin J. Midwives perspectives of their training and education requirements in maternal obesity: a qualitative study. Midwifery. 2013;29(7):736-44.

30. Sanders J, Hunter B, Warren L. A wall of information? Exploring the public health component of maternity care in England. Midwifery. 2016;34:253-60.

31. Butterfield YC, O’Connell B, Phillips D. Peripartum urinary incontinence: A study of midwives’ knowledge and practices. Women and Birth. 2007;20(2):65-9.

32. Doi L, Cheyne H, Jepson R. Alcohol brief interventions in Scottish antenatal care: a qualitative study of midwives' attitudes and practices. BMC Pregnancy & Childbirth. 2014;14:170.

33. McNeill J, Doran J, Lynn F, Anderson G, Alderdice F. Public health education for midwives and midwifery students: a mixed methods study. BMC Pregnancy & Childbirth. 2012;12:142.

34. Hunter B, Sanders J, Warren L. Exploring the Public Health Role of Midwives and Maternity Support Workers: Final Report. Cardiff: Cardiff University, 2015 25 February 2015. Report No.

35. Lee DJ, Haynes CL, Garrod D. Exploring the midwife's role in health promotion practice. British Journal of Midwifery. 2012;20(3).

36. Dessie SG, Hacker MR, Dodge LE, Elkadry EA. Do Obstetrical Providers, Counsel Women About Postpartum Pelvic Floor Dysfunction? Journal of Reproductive Medicine. 2015;60(5-6):205-10.

37. Frawley H, Chiarelli P, Gunn J. Uptake of antepartum continence screening and pelvic floor muscle exercise instruction by maternity care providers: An implementation project. Neurourology and Urodynamics. 2014;33 (6):976-7.

38. Herberts C, Sykes C. Midwives' perceptions of providing stop-smoking advice and pregnant smokers' perceptions of stop-smoking services within the same deprived area of London. Journal of Midwifery & Women's Health. 2012;57(1):67-73.

39. Gillard S, Shamley D. Factors motivating women to commence and adhere to pelvic floor muscle exercises following a perineal tear at delivery: the influence of experience. Journal of the Association of Chartered Physiotherapists in Women's Health. 2010.

40. Whitford HM, Jones M. An exploration of the motivation of pregnant women to perform pelvic floor exercises using the revised theory of planned behaviour. British Journal of Health Psychology. 2011;16(4):761-78.
