# Supplementary material for: Implementing pelvic floor muscle training in women's childbearing years: A critical interpretive synthesis of individual, professional, and service issues
Source: Neurourol Urodyn. 2019 Dec 17;39(2):863–70. doi: 10.1002/nau.24256 (PMC7079154; doi:10.1002/nau.24256)
Supplement: Supplementary file 3 — Supplementary information [file NAU-39-863-s003.docx]

**Opportunities and constraints in formation and shaping of knowledge and understanding about PFMT**

| **Perspective** | **Synthetic constructs: Challenges/concerns** | **Data source** | **Synthetic constructs: Opportunities/recommendation** | **Data source** |
| --- | --- | --- | --- | --- |
| **Women** | Limited knowledge about PFM/PFD/PFMT: What is UI/PFD? What causes UI? What are PFMs and what is their function? What are PFMEs?  Relationship between PFMT and UI/PFD not clearly understood  PFD viewed as a normal consequence of childbirth by women, HCPs, friends, relatives | (1-11)  (1, 3, 4, 8, 10, 12-15) | Women would like to receive consistent, good quality, professional information from a range of reliable sources  Include information about UI, potential consequences of UI and the role of AN PFMT, its benefits and effectiveness for UI prevention  Raise aware of other high-quality, accurate information resources about UI and PFMT, e.g. leaflets, web-based educational materials | (3, 7, 8, 10, 11, 15-19) |
|  | Unaware of benefits/effectiveness of PFMT  Some women lack awareness of treatment options for PFD  Lack of public awareness of UI within ethnic minority communities | (1, 2, 5, 10, 17, 18, 20) |  |  |
|  | Poor body self-perception/awareness may make it difficult to understand PFMC/PFME | (21) | Encourage greater body awareness to improve understanding of PFM and how to perform correct PFMC and PFMT | (21) |
|  | Unsure how to perform effective PFMT | (1-5, 10, 14, 16-18, 22, 23) | Understand proper technique and develop practical skills for PFMC and PFMT, what to do and how to do it | (4, 10, 16, 17, 23, 24) |
|  | Accuracy and quality of information from different sources is variable  Variable quality of information sourced from books, magazines, internet  Leaflets alone are unsatisfactory | (1, 4, 9, 10, 16, 17, 22, 23) | Develop/provide access to high-quality, accurate educational resources for women, including access to multi-media education resources to meet a variety of individual needs | (2, 16, 17, 25) |
|  | Limited resources available for supporting PFMT for ethnic minority groups, e.g. written information in other languages, use of interpreters | (26, 27) | Develop/provide culturally specific resources, e.g. via talk-based media | (1, 26) |
| **HCPs** | Lack of HCP knowledge or insufficient information regarding PFD | (2, 11, 25, 28) | Improve awareness and understanding of PFD through education and training  Receive formal training and education about health promotion topics | (2, 9, 11, 14, 22, 25, 29, 30) |
|  | Belief that PFD is part of normal pregnancy/delivery  Negative impact of popular media representation of public health messages and pregnancy that does not reflect reality | (25)  (31) | *No data specific to opportunities for HCPs related to this construct* |  |
|  | Lack of skills/confidence in skills for assessing PFMC and teaching PFMT | (2, 22, 32) | Identify PFMC assessment procedure acceptable to HCPs, for example, a stepwise approach: “from individualised, detailed verbal instruction, modified according to the pregnant woman’s feedback to the instruction, to visual perineal assessment, to per vaginum PFM examination only if the midwife felt this was necessary” (p.977, (32)  Suggestions for training to improve skills and confidence for health promotion/PFMT include:   - Behaviour change and motivational interviewing techniques - Communication, empathy and support - Understanding socio-economic, ethnic and cultural factors - Focus on ‘how’ to support behaviour change, rather than ‘what’ - Encourage interaction and reflection, with opportunity for midwives to explore own attitudes towards a topic - Use of role play/role modelling to practice skills - Regular updates and peer support | (32)  (6, 27, 29-36) |
|  | Inconsistent or lack of training in PFMT, communication skills for sensitive topics and other public health topics | (2, 27, 30, 35) | Ensure midwives are trained to sufficient standard to provide accurate advice and support for UI and PFMT in AN care  Work with local specialist HCPs, e.g. physiotherapists, continence advisors, to identify opportunities to deliver training | (2, 36, 37) |
|  | Online training lacks interactive element to support skill development | (34) | Provide time and support for HCPs to attend training and deliver learning in practice | (30) |
|  | Making assumptions about women’s knowledge/awareness/beliefs – may mean that information is not offered to women | (29, 30, 35) | Inform and support women to understand about PFD/UI and PFMT, support informed choices about undertaking, adopting and maintaining PFMT  Teach individually-tailored, evidence-based PFMT programmes | (4-6, 13, 16) |
|  | Limited/no evaluation of women’s knowledge or learning following information provision | (38) | *No data mapped to this construct* |  |
|  | Lack of pre-pregnancy public health education for women – all left to midwives to impart knowledge | (27, 31) | *No data mapped to this construct* |  |
| **Service/ Organisation/ Policy** | Lack of funding available for training  Training prioritised by mandatory requirements | (27, 29, 31, 34) | Support recommendations for practice in RCM/CSP joint statement on PFME  Provide time and support for HCPs to attend training and deliver learning in practice  Review and update post-registration midwifery/HCP training in public health related topics  Develop or signpost to multi-media educational and training resources for HCPs, for example:   - Online training for teaching PFMT, e.g. RCM i-learning platform - Patient information leaflets, e.g. from NHS Choices, CSP or NCT website - Online videos teaching PFMT - Professional training pack | (28, 33, 37) |
|  | *No data for organisational challenges/concerns mapped to this construct* |  | Evaluate effectiveness of training to support midwives with delivery of health promotion interventions | (29) |

*AN=antenatal; HCP=healthcare professional; PFD=pelvic floor dysfunction; PFM=pelvic floor muscle; PFMC=pelvic floor muscle contraction; PFME=pelvic floor muscle exercise; PFMT=pelvic floor muscle training; UI=urinary incontinence*

References

1. Doshani A, Pitchforth E, Mayne CJ, Tincello DG. Culturally sensitive continence care: a qualitative study among South Asian Indian women in Leicester. Family Practice. 2007;24(6):585-93.

2. Logan K. Audit of advice provided on pelvic floor exercises. Professional Nurse. 2001;16(9).

3. Buurman MBR, Lagro-Janssen ALM. Women's perception of postpartum pelvic floor dysfunction and their help-seeking behaviour: a qualitative interview study. Scandinavian Journal of Caring Sciences. 2013;27(2):406-13.

4. Chiarelli P, Cockburn J. The development of a physiotherapy continence promotion program using a customer focus. Australian Journal of Physiotherapy. 1999;45(2):111-9.

5. Gillard S, Shamley D. Factors motivating women to commence and adhere to pelvic floor muscle exercises following a perineal tear at delivery: the influence of experience. Journal of the Association of Chartered Physiotherapists in Women's Health. 2010.

6. Hay-Smith EJC, Dean S, Burgio K, McClurg D, Frawley H, Dumoulin C. Pelvic floor muscle training adherence 'modifiers': A review of primary qualitative studies. ICS State of the Science Seminar research paper II of IV. Neurourology and Urodynamics. 2015;34(7):622-31.

7. Mason L, Glenn S, Walton I, Hughes C. Do women practise pelvic floor exercises during pregnancy or following delivery? Physiotherapy. 2001;87(12):662-70.

8. Moossdorff-Steinhauser HFA, Albers-Heitner P, Weemhoff M, Spaanderman MEA, Nieman FHM, Berghmans B. Factors influencing postpartum women's willingness to participate in a preventive pelvic floor muscle training program: A web-based survey. European Journal of Obstetrics Gynecology and Reproductive Biology. 2015;195:182-7.

9. Whitford HM, Alder B, Jones M. A cross-sectional study of knowledge and practice of pelvic floor exercises during pregnancy and associated symptoms of stress urinary incontinence in North-East Scotland. Midwifery. 2007;23(2):204-17.

10. Cooper H, Carus C. Factors affecting women’s adherence with pelvic floor muscle exercises in a first pregnancy: a qualitative interview study. 2015.

11. Hermansen IL, O'Connell B, Gaskin CJ. Are postpartum women in denmark being given helpful information about urinary incontinence and pelvic floor exercises? Journal of Midwifery & Women's Health. 2010;55(2):171-4.

12. Chiarelli P, Campbell E. Incontinence during pregnancy. Prevalence and opportunities for continence promotion. Australian & New Zealand Journal of Obstetrics & Gynaecology. 1997;37(1):66-73.

13. Chiarelli P, Murphy B, Cockburn J. Acceptability of a urinary continence promotion programme to women in postpartum. BJOG: An International Journal of Obstetrics & Gynaecology. 2003;110(2):188-96.

14. Herron-Marx S, Williams A, Hicks C. A Q methodology study of women's experience of enduring postnatal perineal and pelvic floor morbidity. Midwifery. 2007;23(3).

15. Mason L, Glenn S, Walton I, Hughes C. Women's reluctance to seek help for stress incontinence during pregnancy and following childbirth. Midwifery. 2001;17(3):212-21.

16. Ismail SI. An audit of NICE guidelines on antenatal pelvic floor exercises. International Urogynecology Journal. 2009;20(12):1417-22.

17. Mason L, Glenn S, Walton I, Hughes C. The instruction in pelvic floor exercises provided to women during pregnancy or following delivery. Midwifery. 2001;17(1):55-64.

18. Ashworth PD, Hagan MT. Some social consequences of non-compliance with pelvic floor exercises. Physiotherapy. 1993;79(7):465-71.

19. Mason L. Evidence-based midwifery in action Guidelines on the teaching of pelvic floor exercises. British Journal of Midwifery. 2001;9(10).

20. Sange C, Thomas L, Lyons C, Hill S. Urinary incontinence in Muslim women. Nursing Times. 2008;104(25):49-52.

21. Sacomori C, Cardoso FL, Vanderlinde C. Pelvic floor muscle strength and body self-perception among Brazilian pregnant women. Physiotherapy. 2010;96(4):337-43.

22. Guerrero K, Owen L, Hirst G, Emery S. Antenatal pelvic floor exercises: A survey of both patients' and health professionals' beliefs and practice. Journal of Obstetrics and Gynaecology. 2007;27(7):684-7.

23. Fine P, Burgio K, Borello-France D, Richter H, Whitehead W, Weber A, et al. Teaching and practicing of pelvic floor muscle exercises in primiparous women during pregnancy and the postpartum period. American Journal of Obstetrics and Gynecology. 2007;197(1):107.e1-.e5.

24. Whitford HM, Jones M. An exploration of the motivation of pregnant women to perform pelvic floor exercises using the revised theory of planned behaviour. British Journal of Health Psychology. 2011;16(4):761-78.

25. Dessie SG, Hacker MR, Dodge LE, Elkadry EA. Do Obstetrical Providers, Counsel Women About Postpartum Pelvic Floor Dysfunction? Journal of Reproductive Medicine. 2015;60(5-6):205-10.

26. Wells M, Wagg A. Integrated continence services and the female Bangladeshi population. British Journal of Nursing. 2007;16(9):516-9.

27. Sanders J, Hunter B, Warren L. A wall of information? Exploring the public health component of maternity care in England. Midwifery. 2016;34:253-60.

28. Butterfield YC, O’Connell B, Phillips D. Peripartum urinary incontinence: A study of midwives’ knowledge and practices. Women and Birth. 2007;20(2):65-9.

29. Heslehurst N, Russell S, McCormack S, Sedgewick G, Bell R, Rankin J. Midwives perspectives of their training and education requirements in maternal obesity: a qualitative study. Midwifery. 2013;29(7):736-44.

30. Lee DJ, Haynes CL, Garrod D. Exploring the midwife's role in health promotion practice. British Journal of Midwifery. 2012;20(3).

31. Hunter B, Sanders J, Warren L. Exploring the Public Health Role of Midwives and Maternity Support Workers: Final Report. Cardiff: Cardiff University, 2015 25 February 2015. Report No.

32. Frawley H, Chiarelli P, Gunn J. Uptake of antepartum continence screening and pelvic floor muscle exercise instruction by maternity care providers: An implementation project. Neurourology and Urodynamics. 2014;33 (6):976-7.

33. Doi L, Cheyne H, Jepson R. Alcohol brief interventions in Scottish antenatal care: a qualitative study of midwives' attitudes and practices. BMC Pregnancy & Childbirth. 2014;14:170.

34. McNeill J, Doran J, Lynn F, Anderson G, Alderdice F. Public health education for midwives and midwifery students: a mixed methods study. BMC Pregnancy & Childbirth. 2012;12:142.

35. Herberts C, Sykes C. Midwives' perceptions of providing stop-smoking advice and pregnant smokers' perceptions of stop-smoking services within the same deprived area of London. Journal of Midwifery & Women's Health. 2012;57(1):67-73.

36. Gerrard J, ten Hove R. RCM/CSP Joint Statement on Pelvic Floor Muscle Exercise: Improving outcomes for women following pregnancy and birth. London: Royal College of Midwives and Chartered Society of Physiotherapy, 2013.

37. McClurg D, Gerrard J, Ten Hove R. Reducing the incidence of incontinence. British Journal of Midwifery. 2015;23(1):17-20.

38. Wilson J, Berlach RG, Hill A-M. An audit of antenatal education facilitated by physiotherapists in Western Australian public hospitals. Australian & New Zealand Continence Journal. 2014;20(2).
