# Supplementary material for: Implementing pelvic floor muscle training in women's childbearing years: A critical interpretive synthesis of individual, professional, and service issues
Source: Neurourol Urodyn. 2019 Dec 17;39(2):863–70. doi: 10.1002/nau.24256 (PMC7079154; doi:10.1002/nau.24256)
Supplement: Supplementary file 4 — Supplementary information [file NAU-39-863-s004.docx]

**Opportunities and constraints for engagement and participation in PFMT**

| **Perspective** | **Synthetic constructs: Challenges/concerns** | **Data source** | **Synthetic constructs: Opportunities/recommendation** | **Data source** |
| --- | --- | --- | --- | --- |
| **Women** | Lack of salience of PFME, lack of awareness of efficacy of exercises, low expectation of benefit, no obvious reward | (1-10) | PFMT is seen as important by women  Women are keen to participate and would recommend PFMT to others | (1-3, 11) |
|  | Lack of interest, minimal perceived threat of UI, particularly if taught PFMT early in pregnancy | (5, 6, 12-15) | Awareness of importance of healthy lifestyle may help support engagement with promoting pelvic floor health | (3) |
|  | Lack of supervised practice of PFMT by HCP | (16) | Women believe supervised PFMT is good practice  Group based PFMT instruction is motivating for some women | (16)  (2) |
|  | Uncertain how to perform PFMT, e.g. unsure of correct PFMC, number/frequency/duration of PFME | (1, 2, 4, 6-8, 11-13, 16-19) | Increase self-efficacy for PFMC and PFMT  Develop knowledge and mastery of PFMC and feelings of self-control with PFMT | (2, 4, 8, 14, 19) |
|  | Difficulty accessing services for PFMT (location, timing, cost) | (3, 4, 10) | Provide access to high-quality, accurate educational resources for women, including access to multi-media education resources to meet a variety of individual needs | (1, 16, 19, 20) |
|  | Difficulty integrating PFMT into daily life:   - perceived time and energy commitment - prioritisation alongside other roles and responsibilities e.g. caring for baby/other children - remembering to do PFMT - difficulty maintaining regular PFMT | (1, 2, 4-8, 10, 11, 13, 16-18) | Frequent reminders/consistent recommendation for PFMT from various sources (HCPs, relatives, friends) is helpful  Intention to perform PFME/participation in PFMT may be influenced by:   - Attitude to new behaviour (PFME)/belief in effectiveness - Subjective norms (views/opinions of others) - Personal experience of PFD/UI sysmptoms - Knowing a peer who has experienced UI - Motivation to prevent PFD/UI - Self-efficacy for new behaviour - Having a cue to exercise - Past performance of PMFE (pre-pregnancy or during/after previous pregnancy) - Access to reliable source of information | (3, 4, 6, 8, 9, 13, 14, 19) |
|  | Factors relating to higher adherence to PFMT:   - older age - non-smoker - less deprived background - higher education level - participation in regular fitness - better general health - experience of UI symptoms or pelvic pain | (3, 4, 6-8, 10, 21, 22) | Use behaviour change techniques to support adoption and maintenance of PFMT, e.g.:   - Receive individual feedback about PFM function - Use prompts/cues to remember to carry out PFMT - Problem-solve setbacks/disruptions to regular PFMT | (4, 6, 13, 16) |
|  | Pain or discomfort, fear of damage to PFM, frustration with PFME | (1, 4, 9, 13) | *No data relating to opportunities for women for this construct* |  |
|  | Non-adherence may result in self-blame and limited help-seeking, belief that beyond help | (2, 4, 18) | *No data relating to opportunities for women for this construct* |  |
| **HCPs** | Limited resources, time constraints, workload pressures, prioritisation:   - staff shortages - busy antenatal appointments - other public health topics to address - expanding midwifery professional role | (1, 11, 20, 23-32) | Address resource requirements to facilitate PFMT in AN care | (20, 33) |
|  | Lack of confidence in knowledge and skills for discussing and teaching unfamiliar topics, lack of expertise in health promotion  Limited knowledge or understanding of rationale for intervention | (27, 28, 30, 31) | Improve confidence with implementing routine PFME assessment/education/training through access to training | (1, 34) |
|  | Limited time to attend training or embed learning in practice | (29, 31, 32) | Support attendance at training in health promotion topics to raise awareness and increase motivation to prioritise implementation in clinical practice. | (34) |
|  | Engagement influenced by attitudes and beliefs | (27, 34) | Training should provide opportunity for midwives/HCPs to explore and challenge attitudes | (34) |
|  | PFMT not seen as part of routine antenatal care | (26) | Midwives’ see it as their responsibility to empower women to manage their health  RCM/CSP initiative recommendations promote implementation of PFMT by midwives | (30)  (24, 33) |
|  | Lack of appreciation of challenges for changing behaviour for women | (28) | Adopt strategies for enabling engagement and participation in regular PFMT and enhancing adherence, e.g.:   - Use communication skills to encourage regular PFMT performance and positive feelings about PFMT - Provide individually tailored messages to support engagement and participation - Use positive modelling to encourage participation by providing examples of others who have had success with PFMT - Help women to identify personal reasons why engaging in PFMT is important for them - Learn techniques for motivating women who may struggle to adhere to PFMT, e.g. smokers, less educated, not regularly exercising - Identify and address barriers to adherence, recognise and acknowledge challenges for implementing PFMT - Regular follow-up to monitor progress and encourage maintenance of PFMT - Use and teach behaviour change techniques to enhance adherence to PFMT | (3, 4, 10, 14, 21, 23) |
|  | Professional culture – midwifery opposition to performing PFMC assessment | (26) | Consider step-wise approach to AN assessment of PFMC:   - Verbal instruction to visual assessment to vaginal PFM examination as required | (26) |
|  | Lack of time to develop relationship with clients may limit HCP’s ability to engage women and encourage participation, especially certain groups of women such as young mothers | (32) | Work in partnership with women and families to increase their self-efficacy to improve pelvic floor health | (33) |
|  | Assumptions about women’s priorities may influence how HCPs engage women/encourage participation in PFMT  Engagement influenced by attitudes and beliefs | (27-29, 34) | Participation may be enhanced by positive feedback from women and belief in clinical value of intervention | (26) |
| **Service/ Organisation/ Policy** | *No data relating to organisational challenges/concerns for this construct* |  | Design a PFMT implementation strategy in collaboration with clinical leaders/local HCPs involved in AN care, to fit local context and clinical priorities  Empower midwives to implement routine AN continence screening and PFME instruction | (26) |
|  | *No data relating to organisational challenges/concerns for this construct* |  | Make use of appropriate behaviour change techniques at policy/organisation/service/individual levels to promote adherence to PFMT | (4) |
|  | Limited time to attend training or embed learning in practice | (29, 31, 32) | Support attendance at training in health promotion topics to raise awareness and increase motivation to prioritise implementation in clinical practice. | (34) |
|  | *No data relating to organisational challenges/concerns for this construct* |  | Agreement from professional bodies to support implementation of PFMT by midwives | (33) |

*AN=antenatal; HCP=healthcare professional; PFD=pelvic floor dysfunction; PFM=pelvic floor muscle; PFMC=pelvic floor muscle contraction; PFME=pelvic floor muscle exercise; PFMT=pelvic floor muscle training; UI=urinary incontinence*

References

1. Logan K. Audit of advice provided on pelvic floor exercises. Professional Nurse. 2001;16(9).

2. Ashworth PD, Hagan MT. Some social consequences of non-compliance with pelvic floor exercises. Physiotherapy. 1993;79(7):465-71.

3. Moossdorff-Steinhauser HFA, Albers-Heitner P, Weemhoff M, Spaanderman MEA, Nieman FHM, Berghmans B. Factors influencing postpartum women's willingness to participate in a preventive pelvic floor muscle training program: A web-based survey. European Journal of Obstetrics Gynecology and Reproductive Biology. 2015;195:182-7.

4. Hay-Smith EJC, Dean S, Burgio K, McClurg D, Frawley H, Dumoulin C. Pelvic floor muscle training adherence 'modifiers': A review of primary qualitative studies. ICS State of the Science Seminar research paper II of IV. Neurourology and Urodynamics. 2015;34(7):622-31.

5. Chiarelli P, Murphy B, Cockburn J. Acceptability of a urinary continence promotion programme to women in postpartum. BJOG: An International Journal of Obstetrics & Gynaecology. 2003;110(2):188-96.

6. Cooper H, Carus C. Factors affecting women’s adherence with pelvic floor muscle exercises in a first pregnancy: a qualitative interview study. 2015.

7. Fine P, Burgio K, Borello-France D, Richter H, Whitehead W, Weber A, et al. Teaching and practicing of pelvic floor muscle exercises in primiparous women during pregnancy and the postpartum period. American Journal of Obstetrics and Gynecology. 2007;197(1):107.e1-.e5.

8. Gillard S, Shamley D. Factors motivating women to commence and adhere to pelvic floor muscle exercises following a perineal tear at delivery: the influence of experience. Journal of the Association of Chartered Physiotherapists in Women's Health. 2010.

9. Mason L, Glenn S, Walton I, Hughes C. Do women practise pelvic floor exercises during pregnancy or following delivery? Physiotherapy. 2001;87(12):662-70.

10. Whitford HM, Alder B, Jones M. A cross-sectional study of knowledge and practice of pelvic floor exercises during pregnancy and associated symptoms of stress urinary incontinence in North-East Scotland. Midwifery. 2007;23(2):204-17.

11. Guerrero K, Owen L, Hirst G, Emery S. Antenatal pelvic floor exercises: A survey of both patients' and health professionals' beliefs and practice. Journal of Obstetrics and Gynaecology. 2007;27(7):684-7.

12. Doshani A, Pitchforth E, Mayne CJ, Tincello DG. Culturally sensitive continence care: a qualitative study among South Asian Indian women in Leicester. Family Practice. 2007;24(6):585-93.

13. Chiarelli P, Cockburn J. The development of a physiotherapy continence promotion program using a customer focus. Australian Journal of Physiotherapy. 1999;45(2):111-9.

14. Whitford HM, Jones M. An exploration of the motivation of pregnant women to perform pelvic floor exercises using the revised theory of planned behaviour. British Journal of Health Psychology. 2011;16(4):761-78.

15. Chiarelli P, Campbell E. Incontinence during pregnancy. Prevalence and opportunities for continence promotion. Australian & New Zealand Journal of Obstetrics & Gynaecology. 1997;37(1):66-73.

16. Mason L, Glenn S, Walton I, Hughes C. The instruction in pelvic floor exercises provided to women during pregnancy or following delivery. Midwifery. 2001;17(1):55-64.

17. Buurman MBR, Lagro-Janssen ALM. Women's perception of postpartum pelvic floor dysfunction and their help-seeking behaviour: a qualitative interview study. Scandinavian Journal of Caring Sciences. 2013;27(2):406-13.

18. Herron-Marx S, Williams A, Hicks C. A Q methodology study of women's experience of enduring postnatal perineal and pelvic floor morbidity. Midwifery. 2007;23(3).

19. Ismail SI. An audit of NICE guidelines on antenatal pelvic floor exercises. International Urogynecology Journal. 2009;20(12):1417-22.

20. Dessie SG, Hacker MR, Dodge LE, Elkadry EA. Do Obstetrical Providers, Counsel Women About Postpartum Pelvic Floor Dysfunction? Journal of Reproductive Medicine. 2015;60(5-6):205-10.

21. Bø K, Owe KM, Nystad W. Which women do pelvic floor muscle exercises six months' postpartum? American Journal of Obstetrics & Gynecology. 2007;197(1).

22. Mason L, Glenn S, Walton I, Hughes C. The relationship between between ante-natal pelvic floor muscle exercises and post-partum stress incontinence. Physiotherapy. 2001;87(12):651-3.

23. Freeman RM. Can we prevent childbirth-related pelvic floor dysfunction? BJOG: An International Journal of Obstetrics & Gynaecology. 2013;120(2):137-40.

24. McClurg D, Gerrard J, Ten Hove R. Reducing the incidence of incontinence. British Journal of Midwifery. 2015;23(1):17-20.

25. Wilson J, Berlach RG, Hill A-M. An audit of antenatal education facilitated by physiotherapists in Western Australian public hospitals. Australian & New Zealand Continence Journal. 2014;20(2).

26. Frawley H, Chiarelli P, Gunn J. Uptake of antepartum continence screening and pelvic floor muscle exercise instruction by maternity care providers: An implementation project. Neurourology and Urodynamics. 2014;33 (6):976-7.

27. Doi L, Cheyne H, Jepson R. Alcohol brief interventions in Scottish antenatal care: a qualitative study of midwives' attitudes and practices. BMC Pregnancy & Childbirth. 2014;14:170.

28. Herberts C, Sykes C. Midwives' perceptions of providing stop-smoking advice and pregnant smokers' perceptions of stop-smoking services within the same deprived area of London. Journal of Midwifery & Women's Health. 2012;57(1):67-73.

29. Hunter B, Sanders J, Warren L. Exploring the Public Health Role of Midwives and Maternity Support Workers: Final Report. Cardiff: Cardiff University, 2015 25 February 2015. Report No.

30. Lee DJ, Haynes CL, Garrod D. Exploring the midwife's role in health promotion practice. British Journal of Midwifery. 2012;20(3).

31. McNeill J, Doran J, Lynn F, Anderson G, Alderdice F. Public health education for midwives and midwifery students: a mixed methods study. BMC Pregnancy & Childbirth. 2012;12:142.

32. Sanders J, Hunter B, Warren L. A wall of information? Exploring the public health component of maternity care in England. Midwifery. 2016;34:253-60.

33. Gerrard J, ten Hove R. RCM/CSP Joint Statement on Pelvic Floor Muscle Exercise: Improving outcomes for women following pregnancy and birth. London: Royal College of Midwives and Chartered Society of Physiotherapy, 2013.

34. Heslehurst N, Russell S, McCormack S, Sedgewick G, Bell R, Rankin J. Midwives perspectives of their training and education requirements in maternal obesity: a qualitative study. Midwifery. 2013;29(7):736-44.
