# Supplementary material for: Implementing pelvic floor muscle training in women's childbearing years: A critical interpretive synthesis of individual, professional, and service issues
Source: Neurourol Urodyn. 2019 Dec 17;39(2):863–70. doi: 10.1002/nau.24256 (PMC7079154; doi:10.1002/nau.24256)
Supplement: Supplementary file 5 — Supplementary information [file NAU-39-863-s005.docx]

**Social and emotional challenges and opportunities**

| **Perspective** | **Synthetic constructs: Challenges/concerns** | **Data source** | **Synthetic constructs: Opportunities/recommendation** | **Data source** |
| --- | --- | --- | --- | --- |
| **Women** | PFD/UI perceived as taboo  PFD/UI invokes feelings of embarrassment, shame, guilt  Reluctant/uncomfortable discussing PFD/UI | (1-11) | Raise public awareness of PFD and its prevention/treatment/management  Challenge beliefs about PFD as an inevitable consequence of pregnancy and childbirth | (1, 7, 8, 10) |
|  | Limited help seeking for PFD/UI  Hope for spontaneous resolution | (1-4, 7, 8, 10, 12, 13) | Encourage help-seeking:  Ask women direct questions about UI to allow opportunity to discuss issues and ask questions | (8, 14-16) |
|  | PFD/UI ignored or normalised as an inevitable consequence of pregnancy and childbirth by some HCPs, family and friends, reinforcing belief that nothing can be done | (1-8) | Educate women about PFD/UI to lessen taboo and embarrassment | (8) |
|  | Fear of ridicule, concerned about views of others  Fear of judgment by HCPs for not adhering to PFMT | (1, 3, 10, 17)  (7, 18) | *No data relating to women’s opportunities/recommendations for this construct* |  |
|  | Do not want to be a burden to the health service, fear of time wasting, PFD/UI symptoms too trivial to bother HCP | (7, 8, 18) | Women, including ethnic minority groups, express a preference for HCPs to take a direct approach to addressing PFD/UI:   - HCPs to ask direct questions and have open discussion as women may be reluctant to raise PFD/UI themselves - Being asked about PFD/UI helps reassure women that is it is something HCPs can help with and there are options for treatment/management | (1-4, 7, 8) |
|  | Specific cultural challenges and concerns identified by ethnic minority women:   - Communication due to language barriers and literacy - Use of interpreters may be problematic, e.g. if they are from the same community this can be uncomfortable, preference for using family members to interpret but may not wish to disclose UI in their presence - Feel that HCP may be embarrassed discussing PFD/UI - Preference for female HCP - Preference for HCP of same ethnicity - Cleanliness is important for prayer for Muslim women in particular therefore UI can affect religious practices - PFD often viewed as taboo restricting discussion and help-seeking in ethnic minority communities | (1-3, 10, 12) | Ethnic minority perspectives:   - need for greater awareness within ethnic minority communities using culturally specific media, e.g. storyline about UI on Asian TV network or radio - demonstrate sensitivity to emotional, social and cultural impact of symptoms, including impact on religious practices - be aware of literacy issues and offer information via talk-based media, e.g. offer small group talks within local community centres - identify community champions within ethnic community to share experiences and break the taboo of PFD | (1, 3, 10) |
| **HCPs** | HCPs believe women may not feel comfortable divulging information about emotive or sensitive topics if they are embarrassed/ashamed | (19) | Educate women about PFD/UI to lessen taboo and embarrassment  Encourage help-seeking:  Ask women direct questions about UI to allow opportunity to discuss issues and ask questions | (8)  (8, 14-16) |
|  | Difficulty discussing emotive or sensitive topics | (19-21) | Training to improve knowledge, awareness and confidence addressing sensitive and emotive topics | (19, 21) |
|  | Language and cultural barriers may further constrain ability to discuss sensitive topics | (22, 23) | *No data relating to HCP opportunities/recommendations for this construct* |  |
|  | Raising emotive/sensitive topics may have negative impact on HCP-woman relationship | (19-22, 24) | Offer reassurance about PFD/UI and adopt a non-judgemental attitude and supportive approach to communication:   - Acknowledge and validate PFD/UI symptoms where applicable - Avoid conveying judgement or inducing guilt or self-blame for past failure/non-adherence to PFMT | (17, 18) |
|  | Rapport between HCP-woman is not established at first appointment therefore difficult to discuss emotive/sensitive topics at booking/first appointment | (19, 21) | Establish quality relationship with woman to facilitate communication and ensure they feel safe/comfortable discussing sensitive topics | (20, 23, 24) |
| **Service/ Organisation/ Policy** | Lack of communication skills training to support HCPs to discuss sensitive topics | (23) | Provide skills training for HCPs to improve confidence with communication, empathy and support when discussing sensitive/emotive topics | (20, 21, 23) |
|  | Inconsistent provision of services for PFME/PFD, e.g. limited attention to PFM health, AN continence screening not routine, limited advice giving regarding PFME/PFD, variable instruction for PFMT, variable quality of instruction | (13, 25-27) | Develop and implement AN continence screening checklist to promote midwives to routinely ask about PFD/UI and provide instruction in PFMT | (7) |

*AN=antenatal; HCP=healthcare professional; PFD=pelvic floor dysfunction; PFM=pelvic floor muscle; PFMC=pelvic floor muscle contraction; PFME=pelvic floor muscle exercise; PFMT=pelvic floor muscle training; UI=urinary incontinence*

References

1. Doshani A, Pitchforth E, Mayne CJ, Tincello DG. Culturally sensitive continence care: a qualitative study among South Asian Indian women in Leicester. Family Practice. 2007;24(6):585-93.

2. Wells M, Wagg A. Integrated continence services and the female Bangladeshi population. British Journal of Nursing. 2007;16(9):516-9.

3. Wilkinson K. Pakistani women's perceptions and experiences of incontinence. Nursing Standard. 2001;16(5):33-9.

4. Buurman MBR, Lagro-Janssen ALM. Women's perception of postpartum pelvic floor dysfunction and their help-seeking behaviour: a qualitative interview study. Scandinavian Journal of Caring Sciences. 2013;27(2):406-13.

5. Chiarelli P, Cockburn J. The development of a physiotherapy continence promotion program using a customer focus. Australian Journal of Physiotherapy. 1999;45(2):111-9.

6. Cooper H, Carus C. Factors affecting women’s adherence with pelvic floor muscle exercises in a first pregnancy: a qualitative interview study. 2015.

7. Herron-Marx S, Williams A, Hicks C. A Q methodology study of women's experience of enduring postnatal perineal and pelvic floor morbidity. Midwifery. 2007;23(3).

8. Mason L, Glenn S, Walton I, Hughes C. Women's reluctance to seek help for stress incontinence during pregnancy and following childbirth. Midwifery. 2001;17(3):212-21.

9. Moossdorff-Steinhauser HFA, Albers-Heitner P, Weemhoff M, Spaanderman MEA, Nieman FHM, Berghmans B. Factors influencing postpartum women's willingness to participate in a preventive pelvic floor muscle training program: A web-based survey. European Journal of Obstetrics Gynecology and Reproductive Biology. 2015;195:182-7.

10. Chaliha C, Stanton SL. The ethnic cultural and social aspects of incontinence - A pilot study. International Urogynecology Journal. 1999;10(3):166-70.

11. Sacomori C, Cardoso FL, Vanderlinde C. Pelvic floor muscle strength and body self-perception among Brazilian pregnant women. Physiotherapy. 2010;96(4):337-43.

12. Sange C, Thomas L, Lyons C, Hill S. Urinary incontinence in Muslim women. Nursing Times. 2008;104(25):49-52.

13. Logan K. Audit of advice provided on pelvic floor exercises. Professional Nurse. 2001;16(9).

14. Aston B. Preventing pelvic floor dysfunction: childbearing women deserve better care. Journal of Family Health Care. 2009;19(5):150-1.

15. Chiarelli P, Campbell E. Incontinence during pregnancy. Prevalence and opportunities for continence promotion. Australian & New Zealand Journal of Obstetrics & Gynaecology. 1997;37(1):66-73.

16. Whitford HM, Alder B, Jones M. A cross-sectional study of knowledge and practice of pelvic floor exercises during pregnancy and associated symptoms of stress urinary incontinence in North-East Scotland. Midwifery. 2007;23(2):204-17.

17. Hay-Smith EJC, Dean S, Burgio K, McClurg D, Frawley H, Dumoulin C. Pelvic floor muscle training adherence 'modifiers': A review of primary qualitative studies. ICS State of the Science Seminar research paper II of IV. Neurourology and Urodynamics. 2015;34(7):622-31.

18. Ashworth PD, Hagan MT. Some social consequences of non-compliance with pelvic floor exercises. Physiotherapy. 1993;79(7):465-71.

19. Doi L, Cheyne H, Jepson R. Alcohol brief interventions in Scottish antenatal care: a qualitative study of midwives' attitudes and practices. BMC Pregnancy & Childbirth. 2014;14:170.

20. Herberts C, Sykes C. Midwives' perceptions of providing stop-smoking advice and pregnant smokers' perceptions of stop-smoking services within the same deprived area of London. Journal of Midwifery & Women's Health. 2012;57(1):67-73.

21. Heslehurst N, Russell S, McCormack S, Sedgewick G, Bell R, Rankin J. Midwives perspectives of their training and education requirements in maternal obesity: a qualitative study. Midwifery. 2013;29(7):736-44.

22. Hunter B, Sanders J, Warren L. Exploring the Public Health Role of Midwives and Maternity Support Workers: Final Report. Cardiff: Cardiff University, 2015 25 February 2015. Report No.

23. Sanders J, Hunter B, Warren L. A wall of information? Exploring the public health component of maternity care in England. Midwifery. 2016;34:253-60.

24. Lee DJ, Haynes CL, Garrod D. Exploring the midwife's role in health promotion practice. British Journal of Midwifery. 2012;20(3).

25. Dessie SG, Hacker MR, Dodge LE, Elkadry EA. Do Obstetrical Providers, Counsel Women About Postpartum Pelvic Floor Dysfunction? Journal of Reproductive Medicine. 2015;60(5-6):205-10.

26. Frawley H, Chiarelli P, Gunn J. Uptake of antepartum continence screening and pelvic floor muscle exercise instruction by maternity care providers: An implementation project. Neurourology and Urodynamics. 2014;33 (6):976-7.

27. Guerrero K, Owen L, Hirst G, Emery S. Antenatal pelvic floor exercises: A survey of both patients' and health professionals' beliefs and practice. Journal of Obstetrics and Gynaecology. 2007;27(7):684-7.
